# Solid State Cross-Linked Polypropylene via Reactive Extrusion: A Scalable Approach

**DOI:** 10.1021/acs.macromol.6c01058

**Published:** 2026-07-02

**Authors:** Jonas J. Perez-Bravo, Javier González-Benito, Jules A. W. Harings

**Affiliations:** † Departamento de Ciencia de Materiales y de Ingeniería Química, 88082 Universidad Carlos III de Madrid, Avda. Universidad 30, Leganés 28911, Spain; ‡ Instituto Tecnológico de Química y Materiales “Álvaro Alonso Barba”, Universidad Carlos III de Madrid, Avda. Universidad 30, Leganés 28911, Spain; § Aachen-Maastricht Institute for Biobased Materials (AMIBM), 5211 Maastricht University, P.O. Box 616, MD Maastricht 6200, The Netherlands

## Abstract

Solid-state reactive extrusion provides a promising route for modifying semicrystalline polymers while minimizing degradation associated with conventional melt processing. Here, isotactic polypropylene was modified using benzoyl peroxide at 110 °C, below the polymer melting temperature. Under these conditions, radical reactions occur within the amorphous regions of the semicrystalline matrix while crystalline lamellae remain intact and restrict large-scale chain motion. FTIR and ^13^C CP-MAS NMR analyses indicate that the polypropylene backbone is largely preserved after processing. Differential scanning calorimetry shows increased crystallization temperatures and reduced crystallinity, consistent with restricted chain mobility and network-induced nucleation. Rheological measurements reveal a progressive increase in the storage modulus with increasing peroxide concentration and a transition toward predominantly elastic behavior with the absence of G’/G” crossover, indicating increased molecular connectivity and network formation. Compared with conventional melt-state peroxide modification, the solid-state approach promotes intermolecular connectivity while limiting degradation pathways, providing an energy-efficient and scalable strategy for tailoring polypropylene properties.

## Graphical abstract

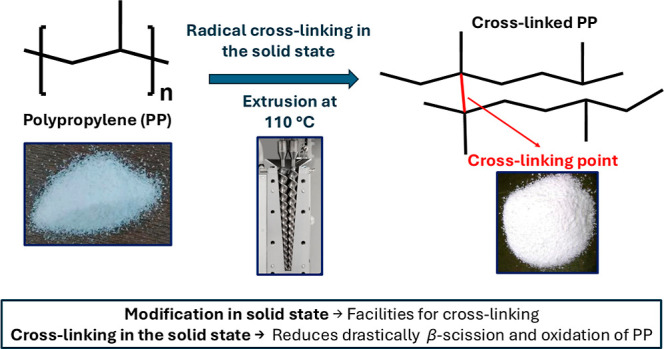

## 1 Introduction

Polypropylene (PP) is widely produced and considered one of the most versatile plastics worldwide.^1^ PP is widely used because it is lightweight, inexpensive, and chemically resistant.^2^ However, PP has weaknesses that limit its use at high temperatures, under long-term load, or in aggressive environments.^3^

Cross-linking is the chemical joining of polymer chains, leading to a three-dimensional network that may address the reported weaknesses of PP.^3,4^ Cross-linking can enhance PP thermal stability, mechanical strength, and chemical and environmental resistance.^5^ Furthermore, partially cross-linked or long-chain-branched PP exhibits higher melt strength and elasticity, thereby improving foamability, film drawability, and thermoforming behavior without compromising recyclability.^6,7^ Once cross-linked, PP resists shrinkage and warpage during service, molding, or exposure to heat, making it useful for precision parts and multilayer composites.^7^

However, PP chemical modification poses significant challenges, particularly when ensuring material integrity during modification.^8^ Traditional chemical modifications of PP are generally carried out in the melt state at temperatures between 160 and 250 °C.^9^ These conditions facilitate oxidation and β-scission of polymer chains, resulting in a considerable reduction in molecular weight, deterioration in mechanical properties, and the formation of oxidative byproducts that contribute to discoloration and the release of volatile emissions.^10^ Consequently, these thermal degradation pathways impose critical limitations on the quality and performance of modified PP, thereby hindering its applicability in high-value applications.^11^

Reactive extrusion has been an attractive strategy for industrial polymer modification, thanks to its solvent-free nature and its integration with existing processing lines.^12^ However, traditional melt-state reactive extrusion of PP with radical initiators often exacerbates chain scission and oxidative degradation, especially under the high shear and thermal stresses inherent to melt processing.^8^ There is therefore a pressing need for new processing approaches that enable efficient chemical modification of PP while minimizing molecular weight loss and preserving material mechanical performance.

To address these limitations, we report a fundamentally disruptive methodology for the chemical modification of polypropylene in the solid state by reactive extrusion using a commercial initiator, benzoyl peroxide (BPO), under conditions well below the polymer’s melting temperature (solid state). In contrast to the melt processing approach, our work is carried out within PP’s solid amorphous domains.^8^ These regions, though with a lower volume fraction than the liquid state, allow sufficient mobility at moderate temperatures to favor controlled diffusion and radical reactions.^8,13^ Simultaneously, the surrounding crystalline lamellae provide dimensional stability and reduce the mobility of macromolecular chains. Consequently, the polymer backbone remains mostly intact, preventing chain scission and maintaining both molecular weight and mechanical integrity.^8,13^

Conventional peroxide-assisted reactive extrusion of polypropylene is typically performed between 180 and 250 °C, where the high mobility of PP macroradicals promotes β-scission, molecular weight reduction, and oxidative degradation.^9,10^ Hamielec et al. and subsequent studies reported that peroxide treatment under melt-processing conditions often results in a competition between chain scission, long-chain branching, and cross-linking. In contrast, the present methodology operates at 110 °C, well below the melting temperature of PP, where crystalline constraints limit chain mobility and are expected to suppress β-scission while favoring radical recombination pathways.^10^ In contrast to melt processing, the present work is conducted within the amorphous regions of a semicrystalline polypropylene matrix while the material remains below its melting temperature. At 110 °C, the amorphous phase is above its glass transition temperature and therefore retains sufficient segmental mobility to enable diffusion and radical reactions, whereas the crystalline lamellae remain intact, providing dimensional stability and topological constraints.^8,14^

From a thermochemical approach, the process exploits the selective generation of radicals at submelting temperatures, minimizing side reactions and enabling a cleaner, more selective functionalization pathway. This solid-state cross-linking strategy offers a range of advantages: significantly minimizing oxidative discoloration and PP chain scission and oxidation, which contribute to lower polymer molecular weight, all within an energy-efficient, solvent-free process compatible with existing industrial extrusion infrastructure. This method opens up new possibilities for producing high-performance polypropylene materials. It implies a significant shift toward sustainable processing of polyolefins and other thermoplastic polymers. Additionally, its scalability and simplicity make it an effective tool for revalorizing postconsumer PP and for the targeted development of advanced functional materials from both virgin and recycled thermoplastic polymers.

## 2 Experimental Section

### 2.1 Chemicals

Isotactic polypropylene (average Mw of 250,000 and Mn of 67,000) was purchased from Sigma-Aldrich (Germany) and used without further purification and as received.

### 2.2 Preparation of Cross-Linked Polypropylene (XLPP)

PP pellets were ground using an MF 10 basic Microfine grinder drive (IKA Werke, Germany) under cryogenic conditions (−196 °C). The grinding was performed in two cycles at a frequency of 30 1/s for 2 min, employing four stainless-steel balls (15 mm in diameter). This procedure yielded a PP powder with an average particle diameter of approximately 250 μm. Subsequently, 3.76 g of PP powder and 0.24 g of Luperox A45 (Sigma-Aldrich) were blended in a twin-screw compounder (Xplorer MC 15HT, Netherlands) at 110 °C and 100 rpm for 15 min to ensure homogeneous mixing and initiate the radical reaction. The resulting material was then extracted and stored for further analysis. XLPP was prepared with different initiator/PP weight percentages of 2/98 wt %, 4/96 wt %, and 6/94 wt %, denoted as PP-2, PP-4, and PP-6, respectively.

### 2.3 Characterization

#### 2.3.1 Solid State Nuclear Magnetic Resonance

The ^13^C­{1H} CP/MAS experiments for samples PP and PP-6 were done at Bruker Avance NEO (11.74 T, νL­(1H) = 500.38 MHz, νL­(13C) = 125.83 MHz) using a 4 mm H/F/X MAS DVT probe equipped with magic-angle gradient coils. 60 mg of chitin and CT-30 pipetted into 4 mm HR-MAS rotors, equipped with an upper spacer made of Teflon, and sealed with a Kel-F screw and cap. Adamantane was used as an external reference for calibration of radiofrequency fields (νrf­(1H) = 50.0 kHz and τπ/2 = 5.0 μs) and referencing the chemical shift scale (δ (1H) = 1.85 ppm and δ (13C) = 29.47 ppm).

#### 2.3.2 Fourier Transform Infrared

Spectroscopy analysis: FTIR spectra of PP and cross-linked samples were recorded in the range of 600–4000 cm^–1^. Samples in powder form were placed directly onto the ATR accessory equipped with a diamond crystal window (GladiATR, PIKE Technologies). Spectra were acquired using a Nicolet iS5 FTIR spectrometer (Thermo Scientific) with a resolution of 4 cm^–1^. To enhance the signal-to-noise ratio, 32 scans were averaged in autoaccumulation mode for each measurement.

#### 2.3.3 Differential Scanning Calorimetry Analysis

The DESs were analyzed using a DSC 2500 (TA, USA). The analyses were conducted under a constant nitrogen stream at a flow rate of 30 mL/min. All samples were tightly sealed in aluminum pans. The temperature range for the analysis varied from −90 to 200 °C, with a heating rate of 10 °C/min. The melting and crystallization points and enthalpies were obtained from the DSC curves using TA TRIOS software.

#### 2.3.4 Thermogravimetric Analysis

TGA was performed using a TGA 5500 (TA, USA). Approximately 10 mg of sample was placed in an open platinum pan and heated from 30 to 600 °C at 10 °C/min under nitrogen (30 mL/min). Mass loss and derivative mass loss (DTG) were recorded as functions of temperature. The onset degradation temperature, maximum degradation rate temperature, and final residual mass were obtained from the TGA curves using TA TRIOS software.

#### 2.3.5 Rheology

Rheometer specimens need to be compression molded. The molten samples were rapidly cooled to room temperature under pressure to prevent crystallization gradients. Rheological measurements: Rheological experiments are conducted on a rotational rheometer (Anton Paar MCR 302) equipped with parallel plates and a Peltier-controlled lower plate. All measurements are performed at 190 °C. Dynamic frequency sweeps are performed within the LVE range over an angular frequency (ω) range of 0.1–100 Hz.

## 3 Results and Discussion

### 3.1 Evidence of PP Cross-Linking via Solid State Employing an Extruder

FTIR spectra of neat PP and PP cross-linked with Luperox A75 with 2–6 wt % (PP-2, PP-4, and PP-6, respectively) display the expected polypropylene backbone features (Figure 1a). The characteristic C–H stretching bands appear at 2950 and 2870 cm^–1^ (CH_3_ asymmetric and symmetric and absorption bands corresponding to the C–H stretching of CH_2_), while the deformation region shows the CH_2_ scissoring at 1454–1456 cm^–1^ and the CH_3_ symmetric deformation at 1375 cm^–1^.^15^ The fingerprint region retains the typical PP peaks at 1160–1000, 973, and 840 cm^–1^, indicating that the polyolefin structure is preserved across formulations.^15^ Under the present solid-state conditions, chain scission and bulk oxidation are not expected because molecular mobility and oxygen diffusion are severely limited within the semicrystalline matrix.^16^ The cross-linking reaction therefore proceeds exclusively through the abstraction of tertiary hydrogens from the polypropylene chain by peroxide-derived radicals, followed by radical recombination between adjacent macroradicals to form C–C junctions.^17,18^

**Figure 1:**
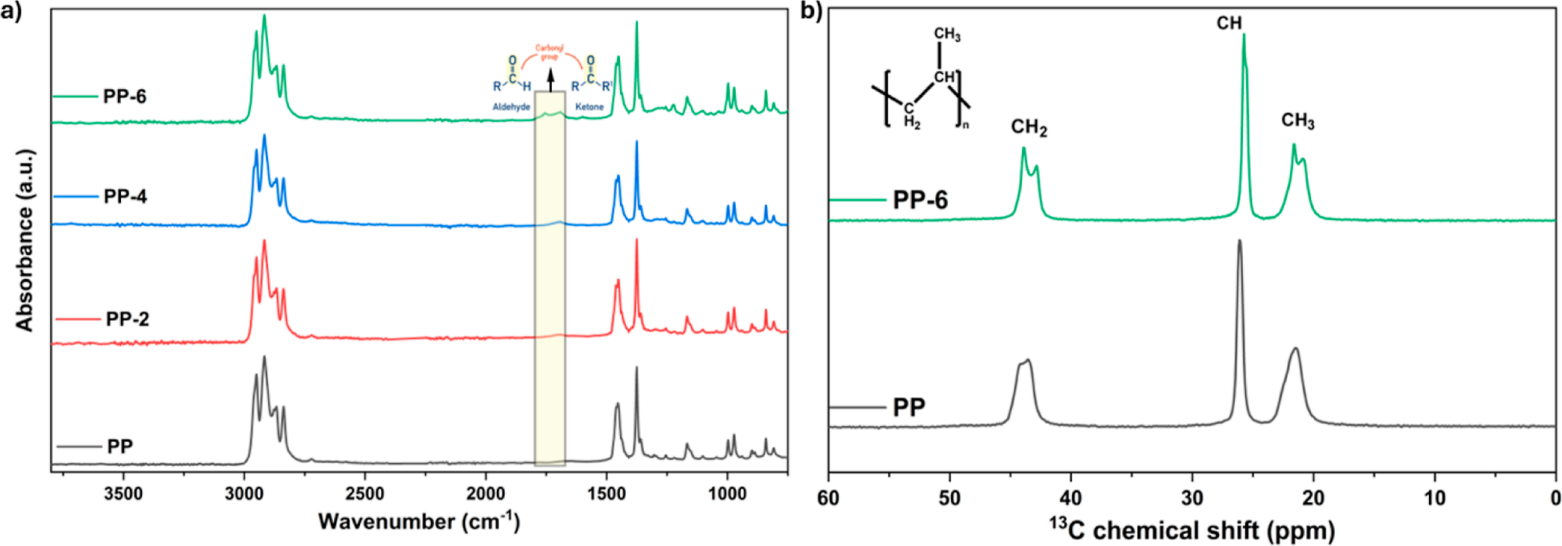
FTIR (a) and ^13^C NMR spectra (b) of pristine and cross-linked polypropylene.

In peroxide-treated samples, weak but reproducible bands assigned to carbonyl emerge in the range 1680–1800 cm^–1^ (maxima near 1686–1692 cm^–1^). These peaks are consistent with mild oxidative products, such as ketones, aldehydes, carboxyls, and/or hydroperoxides, that can be produced during initiator degradation under an oxidizing atmosphere (air).^19,20^ It has been reported that acetone, acetic acid, 2,4-pentanedione, acetaldehyde, formaldehyde, and methyl acrolein can facilitate the oxidation of polypropylene (PP) in the solid state at 140 °C.^21^ In our study, the spectra show clearer, more intense absorption bands, which can be attributed to the higher initiator concentration. This observation indicates that the initiator plays a dominant role in driving the formation of oxygen-containing organic species. Therefore, the FTIR results support (i) the successful initiation of radical chemistry under the selected solid-state conditions, (ii) peroxide-dependent oxidation in the presence of air, and (iii) the occurrence of a maximum in normalized carbonyl formation.

^13^C CP-MAS NMR spectroscopy was employed to probe the structural and morphological changes induced by peroxide-mediated reactive extrusion of polypropylene (Figure 1b). The spectra of both neat PP and cross-linked samples (e.g., PP-6) are dominated by the characteristic resonances of isotactic polypropylene, corresponding to the methylene (44–45 ppm), methine (28 ppm), and methyl (23 ppm) carbons, indicating that the primary hydrocarbon backbone remains largely intact after processing. The absence of significant chemical shift changes confirms that cross-link formation occurs at relatively low concentrations and does not substantially alter the average chemical environment of the repeating units. This observation is consistent with previous solid-state NMR studies of polypropylene, which show that the dominant spectral features remain governed by the semicrystalline backbone even upon chemical modification or degradation.^22^

Despite the preservation of peak positions, noticeable differences in line shape and relative intensity are observed before and after the use of peroxide initiator. In CP-MAS experiments, signal intensity is strongly dependent on segmental mobility and dipolar coupling efficiency; therefore, the spectra are inherently biased toward rigid domains. In semicrystalline polypropylene, this leads to preferential detection of crystalline and rigid amorphous fractions, while highly mobile amorphous segments contribute less efficiently. Consequently, the spectral changes observed for PP-6 are attributed primarily to an increase in constrained amorphous and interfacial regions arising from cross-link formation, which reduces local chain mobility and enhances the relative contribution of rigid domains to the CP signal. Such redistribution of intensity between amorphous and crystalline resonances has been directly observed in solid-state 13C CP-NMR studies of polypropylene subjected to degradation or irradiation.^22^

Importantly, such chemical modifications are preferentially excluded from well-developed crystalline lamellae and are instead localized in amorphous or interfacial regions. As a result, the crystalline domains largely retain the chemical signature of isotactic polypropylene, while the amorphous fraction becomes increasingly constrained and chemically heterogeneous. This phase-selective localization explains why the primary effects of cross-linking and oxidation are reflected in peak broadening and intensity redistribution rather than in the formation of entirely new dominant resonances. Similar behavior has been reported for polypropylene subjected to irradiation or long-term oxidative aging, where the buildup of defect structures primarily affects the amorphous phase while preserving the crystalline lattice.^23,24^

In addition to cross-linking, peroxide treatment is known to generate radicals and oxygen-containing organic species from the initiator. Solid-state ^13^C CP-MAS NMR investigations of oxidized polypropylene using isotopically labeled samples have demonstrated that most chemical modifications originate from reactions at the tertiary carbon sites, leading to the formation of peroxides, alcohols, ketones, and carboxyl-containing species. These oxidation products are typically present at low concentrations and distributed across a range of chemical environments, resulting in weak, often unresolved resonances in natural-abundance CP-MAS spectra.^24^ However, their presence is consistent with complementary spectroscopic evidence (FTIR carbonyl absorption) and contributes to increased structural heterogeneity in the amorphous phase.

^13^C CP-MAS results support a structural model in which peroxide-induced reactive extrusion generates a heterogeneous network consisting of intact polypropylene crystalline lamellae embedded within a progressively constrained and partially cross-linked amorphous phase. The observed spectral changes therefore arise from the combined effects of reduced chain mobility, increased rigid-amorphous fraction, and the formation of low concentrations of chemically modified sites.

Contrasting conventional melt-state peroxide modification, where the formation of unsaturated species and chain-scission products is frequently reported, the present spectra do not show evidence of extensive backbone degradation. This observation suggests that the restricted mobility of the semicrystalline matrix limits degradation pathways while allowing localized radical recombination within amorphous regions.

### 3.2 Thermal Stability and Decomposition Behavior

TGA and DTG (Figure 2a,b) were performed to evaluate the effect of solid-state cross-linking on the thermal stability of polypropylene (Figure 2a,b). All samples exhibit the characteristic single-step thermal decomposition profile of polypropylene, with the main degradation event occurring between 380 and 480 °C.^11,25^ This decomposition window corresponds to chain scission initiated at tertiary carbons, followed by rapid depolymerization to volatile hydrocarbons, a mechanism well established for isotactic PP under inert atmospheres.^25,26^

**Figure 2:**
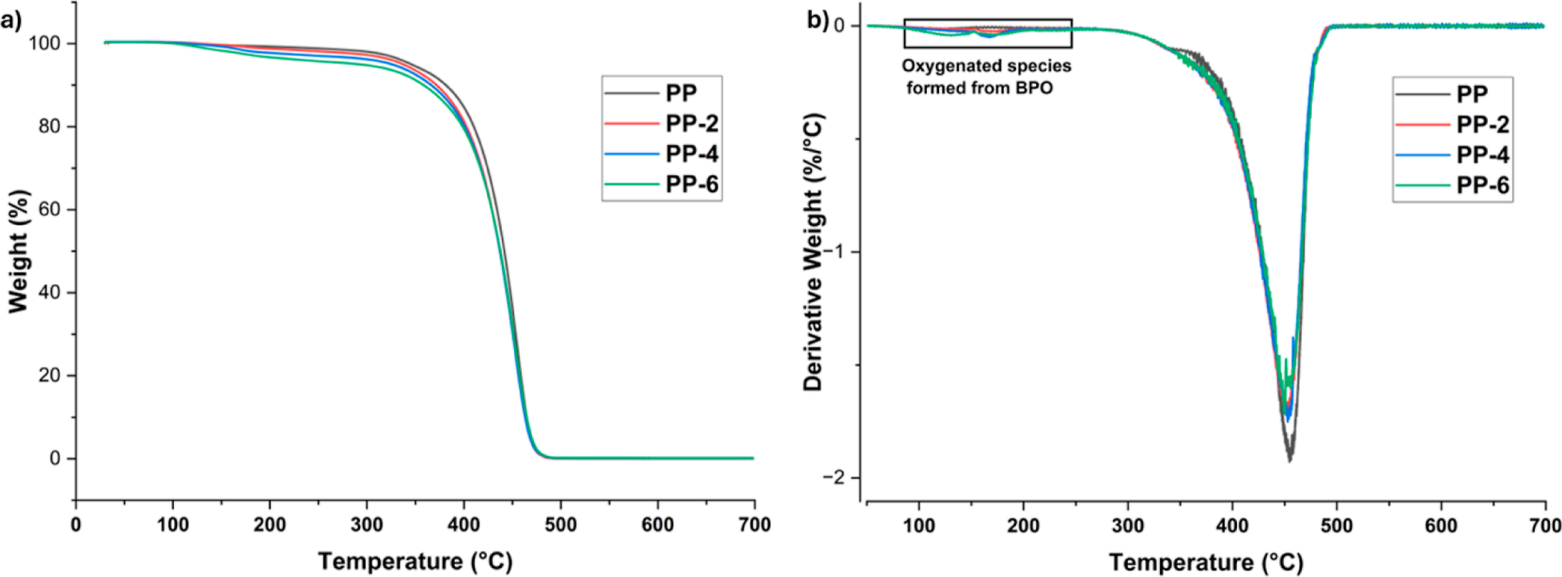
Thermal degradation behavior of pristine and cross-linked polypropylene by TGA (a) and DTG analysis (b).

The onset degradation temperature (*T*_onset_) decreases with increasing initiator concentration: PP > PP-2 > PP-4 > PP-6 (Table 1). This reduction in *T*_onset_ arises from the presence of low-molecular-weight volatile fragments generated during BPO decomposition, which volatilize at lower temperatures and trigger early mass loss. Similar decreases in *T*_onset_ have been reported in peroxide-modified polyolefins, where initiator fragments and oxygenated species formed during radical initiation introduce thermally labile sites.^4^ ^,^ ^6^ This behavior is consistent with the FTIR results, which show weak carbonyl–OH bands arising from initiator-derived oxidation products rather than PP backbone oxidation.

**Table 1:** Thermogravimetric Parameters of PP and Cross-Linked PP Samples

sample	*T*_onset_ (°C)	*T*_5%_ (°C)	residue at 600 °C (%)	*T*_max_ (°C)
PP	287.90	343.00	0.04	454.49
PP-2	237.30	334.00	0.10	452.87
PP-4	226.90	320.60	0.15	452.14
PP-6	200.00	281.30	0.23	449.80

Likewise, the temperature corresponding to 5% mass loss (*T*_5%_) decreases from 343 °C (PP) to 281.3 °C (PP-6), also points out that early mass loss is mainly influenced by peroxide-derived species rather than by polypropylene degradation. These volatiles are not covalently bound to the polymer matrix and are expected to fully dissipate during melt processing or extrusion, ensuring that no residual peroxide species remain in the final cross-linked product.

Despite differences in *T*_onset_ and *T*_5%_, all samples exhibit similar maximum degradation temperatures (*T*_max_), ranging from 449.8 to 454.5 °C. Because *T*_max_ corresponds to the temperature at which the maximum degradation rate of the PP backbone, chemical integrity of the polymer chain is preserved during solid-state cross-linking. This observation agrees with the ^13^C CP-MAS NMR data, which show no unsaturated or carbonyl carbons in the polymer backbone, and with the FTIR spectra, which preserve all major PP fingerprint bands. These results confirm that the cross-linking reaction proceeds via macroradical recombination in the solid state.

A progressive increase in high-temperature residue is also observed, from 0.04% (PP) to 0.23% (PP-6). Although modest, this increase is consistent with the formation of C–C cross-link junctions, which improve char stability by reducing the fraction of fully volatile species during thermal decomposition. Similar increases in char yield have been reported for peroxide-cross-linked polyolefins and other lightly cross-linked PP systems, in which covalent network formation yields a thermally more persistent residue.^4,27^

The TGA/DTG analysis demonstrates that the solid-state cross-linking procedure (i) introduces a small amount of volatile peroxide-derived byproducts that reduce *T*_onset_ and *T*_5%_, but (ii) preserves the intrinsic thermal stability of the polypropylene backbone, as evidenced by the unaltered *T*_max_ values and single-step degradation behavior. This is fully consistent with the spectroscopic evidence (FTIR and ^13^C CP-MAS), which reinforces the idea that the polymer structure remains intact and that cross-linking proceeds without oxidative deterioration.

DSC was employed to evaluate the effect of solid-state cross-linking on the thermal transitions and crystallinity of polypropylene (Table 1). All samples exhibit two well-defined melting endotherms (Figure 4) and crystallization exotherms, confirming that the semicrystalline character of PP is preserved after the processing considered in the solid state.

On second heating (Figure 3a), the melting temperature (*T*_m_) decreases, and the presence of two endothermic peaks is generally attributed to the melting of two distinct crystalline populations.^28^ These peaks can be explained by the coexistence of crystals with varying sizes, degrees of perfection, and thermal stabilities.^29^ The lower-temperature melting peak is associated with the melting of smaller, thinner, and less perfect lamellae, which typically form in constrained environments. Such crystals are often located in regions affected by cross-linking, branching, or local network formation, where chain mobility during crystallization is reduced. In contrast, the higher-temperature melting peak corresponds to the melting of larger, thicker, and more perfect lamellae formed by less constrained, bulk-like polypropylene chains that are able to crystallize more efficiently. This type of bimodal melting behavior has been widely reported for cross-linked polypropylene, long-chain branched polypropylene, and chemically modified polyolefins. However, an alternative explanation for the appearance of two endothermic peaks involves melting and recrystallization phenomena. During heating, part of the less stable crystalline fraction may melt first and subsequently reorganize or recrystallize into more stable crystals, which then melt at higher temperatures. This melting–recrystallization-remelting process can enhance the separation between the two endothermic events, contributing to the observation of two distinct melting peaks. On the other hand, a systematic change in the peak intensity ratio is observed with increasing initiator content from 157.5 °C (PP) to 150.2 °C (PP-6), which can be explained by the corresponding increase in cross-link density. As the initiator concentration increases, the fraction of constrained chain segments rises due to the formation of additional cross-links (Table 2), leading to a progressive reduction in chain mobility and folding ability. Consequently, the low-temperature melting peak increases in intensity, reflecting the larger fraction of imperfect, constrained crystals, while the high-temperature melting peak decreases because the formation of large, well-organized lamellae from bulk-like chains is progressively suppressed with increasing initiator content.

**Figure 3:**
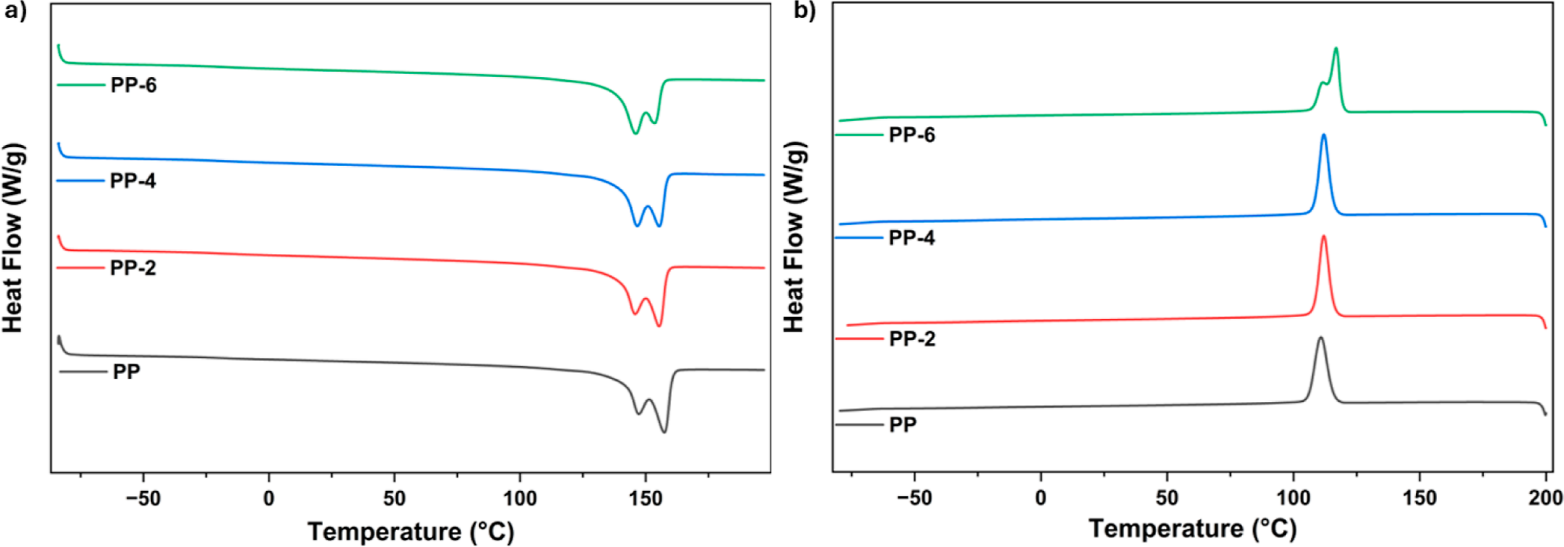
Melting (a) and crystallization (b) behavior of PP and cross-linked PP samples by DSC.

**Table 2:** Melting and Crystallization Temperatures, along with their Enthalpies and the Degree of Crystallinity, were Measured Using DSC

sample	*T*_m_ (°C)	*T*_c_ (°C)	Δ*H*_m_ (J/g)	Δ*H*_c_ (J/g)	crystallinity Xc (%)
PP	157.50	110.85	77.50	84.35	37.45
PP-2	155.40	112.02	72.13	79.23	34.84
PP-4	154.22	112.87	70.67	78.40	34.14
PP-6	150.17	116.87	67.87	77.97	32.79

It is accompanied by a reduction in melting enthalpy (ΔH_m_) from 77.5 J g^–1^ to 67.9 J g^–1^. These trends reflect the formation of thinner or more imperfect lamellae when cross-links partially restrict chain rearrangement, and the subsequent lamellar thickening during recrystallization. The calculated crystallinity (*X*_c_), derived from ΔH_m_/Δ*H*°_m_ (PP) with Δ*H*°_m_ = 207 J g^–1^, decreases from 37.5% for neat PP to 32.8% for PP-6.

During cooling (Figure 3b and Table 2) from the melt, the crystallization peak temperature (*T*_c_) increases progressively from 110.9 °C for neat PP to 116.9 °C for PP-6, suggesting that peroxide-induced cross-linking promotes crystallization by enhancing nucleation efficiency. This upward shift is attributed to covalent junctions and oxidized radicals formed during peroxide decomposition, which can serve as heterogeneous nucleation sites and accelerate the ordering of adjacent chain segments. The corresponding crystallization enthalpies (ΔH_c_) decrease from 84.35 J g^–1^ to 77.97 J g^–1^, suggesting that although crystallization begins earlier (Table 2), the overall crystallizable fraction decreases slightly as cross-links constrain chain mobility within amorphous domains.

Several studies have focused on the cross-linking of polypropylene. These works compare linear isotactic polypropylene (i-PP) with long-chain-branched polypropylene (LCB-PP). They demonstrate that LCB-PP crystallizes at a higher temperature than linear PP, attributed to an increased nucleation density.^30,31^ As a result, a more branched or cross-linked structure raises the Tc and accelerates the overall crystallization process, while slightly lowering the melting temperature (*T*_m_) and crystallinity. These findings are consistent with our research.

The combined calorimetric behavior shows that solid-state cross-linking creates a structurally integrated yet slightly less mobile polymer network. Crystallization begins at higher temperatures due to network-induced nucleation. During recrystallization, restricted molecular motion results in a slight decrease in lamellar perfection and overall crystallinity.

### 3.3 Rheological Evidence of Network Formation

To further evaluate the effect of peroxide-induced modification on the viscoelastic properties of polypropylene and provide additional evidence of network formation, oscillatory rheological measurements were performed (Figure 4). Neat PP exhibits the characteristic rheological behavior of a linear thermoplastic melt. At low frequencies, the loss modulus (G″) exceeds the storage modulus (G′), indicating that viscous dissipation dominates the mechanical response.^32^ As the frequency increases, a crossover between G′ and G″ is observed, reflecting the transition from predominantly viscous to increasingly elastic behavior due to chain entanglements and reduced relaxation times.^33^

**Figure 4:**
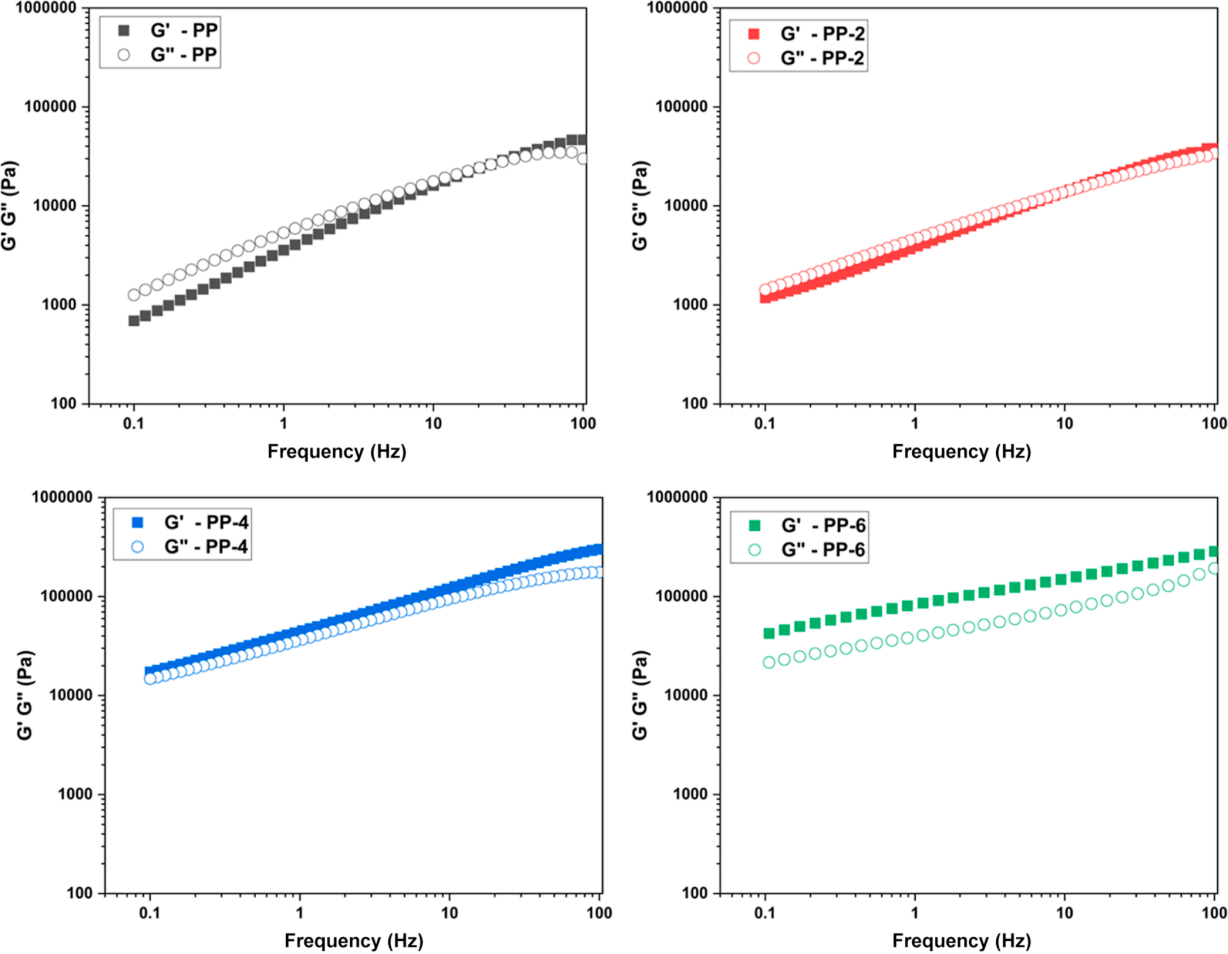
Storage modulus (G′) and loss modulus (G″) as a function of angular frequency of pristine PP and modified PP in solid state at 190 °C (PP-2, PP-4, and PP-6).

Upon incorporation of peroxide, a progressive increase in the elastic response is observed. For PP-2, both moduli increase relative to neat PP, while the crossover between G′ and G″ becomes less pronounced, indicating the onset of intermolecular connections that restrict molecular mobility. This behavior suggests the formation of a lightly connected structure within the polymer matrix. More significant changes are observed for PP-4 and PP-6. In these samples, the storage modulus remains higher than the loss modulus throughout the entire frequency range investigated, and no crossover point is detected. Such behavior is characteristic of materials with network-like structures, in which long-range molecular connectivity suppresses terminal flow and enhances elastic energy storage. The substantial increase in G′, particularly at low frequencies, indicates a marked reduction in chain relaxation and is consistent with the formation of intermolecular covalent junctions generated during peroxide treatment.^34^

The progressive increase in both G′ and G″ with increasing peroxide concentration further demonstrates that the modification process affects the molecular architecture of polypropylene. While the increase in G″ reflects greater resistance to molecular motion, the more pronounced increase in G′ indicates that elastic contributions become increasingly dominant as peroxide concentration increases. This trend is consistent with the development of a partially cross-linked network within the amorphous regions of the semicrystalline polypropylene matrix.

These rheological observations complement the FTIR, ^13^C CP-MAS NMR, DSC, and TGA results. While the spectroscopic analyses indicate preservation of the polypropylene backbone and the thermal analyses reveal changes in crystallization behavior consistent with restricted chain mobility, rheology provides direct evidence of changes in melted viscoelasticity associated with network formation. The absence of a G’/G″ crossover for PP-4 and PP-6, together with the substantial enhancement of the storage modulus, supports the conclusion that peroxide treatment promotes the formation of a network-like structure through macroradical recombination during solid-state reactive extrusion. The rheological results provide strong independent evidence that the solid-state reactive extrusion process generates progressively higher levels of molecular connectivity as peroxide concentration increases, supporting the proposed mechanism of peroxide-induced cross-linking in polypropylene while maintaining the integrity of the polymer backbone.^35^

Despite these similarities, several important differences distinguish the present work from conventional peroxide-cross-linked polypropylene systems. First, most literature studies perform cross-linking under melt-state conditions, typically between 180 and 250 °C, where polymer chains are highly mobile and peroxide-derived radicals can induce both cross-linking and extensive β-scission. As a result, melt-state peroxide modification often leads to competition among branching, cross-linking, and chain degradation. In contrast, the present work employs solid-state reactive extrusion at 110 °C, below the melting temperature of polypropylene. Under these conditions, chain mobility is significantly restricted by the semicrystalline structure, which is expected to suppress β-scission and favor radical recombination within amorphous regions. Consequently, the observed increase in elasticity is accompanied by no significant evidence of backbone degradation, as supported by FTIR, ^13^C CP-MAS NMR, TGA, and DSC analyses.

Second, conventional highly cross-linked polyolefins often exhibit a rheological plateau at low frequencies, indicating a permanent network and near-complete suppression of molecular relaxation. In the present study, although PP-4 and PP-6 exhibit *G*′ > *G*″ across the entire frequency range, both moduli remain frequency-dependent and continue to increase with frequency. This suggests that the material does not behave as a fully cross-linked thermoset network but rather as a partially cross-linked or highly constrained network structure. This distinction is particularly important because it indicates that the peroxide treatment generates sufficient intermolecular connectivity to significantly alter the viscoelastic response while still preserving the processability of polypropylene.^35^

### 3.4 Mechanism of Cross-Linking in the Solid State

Figure 5 illustrates the solid-state radical cross-linking mechanism of PP initiated by BPO. When BPO is heated, the BOP O–O bond undergoes homolytic cleavage, producing two benzoyloxy radicals. These highly unstable radicals rapidly decarboxylate, generating phenyl radicals and carbon dioxide, as shown in the second panel of Figure 4. Both benzoyloxy and phenyl radicals serve as potent hydrogen-abstracting species.

**Figure 5:**
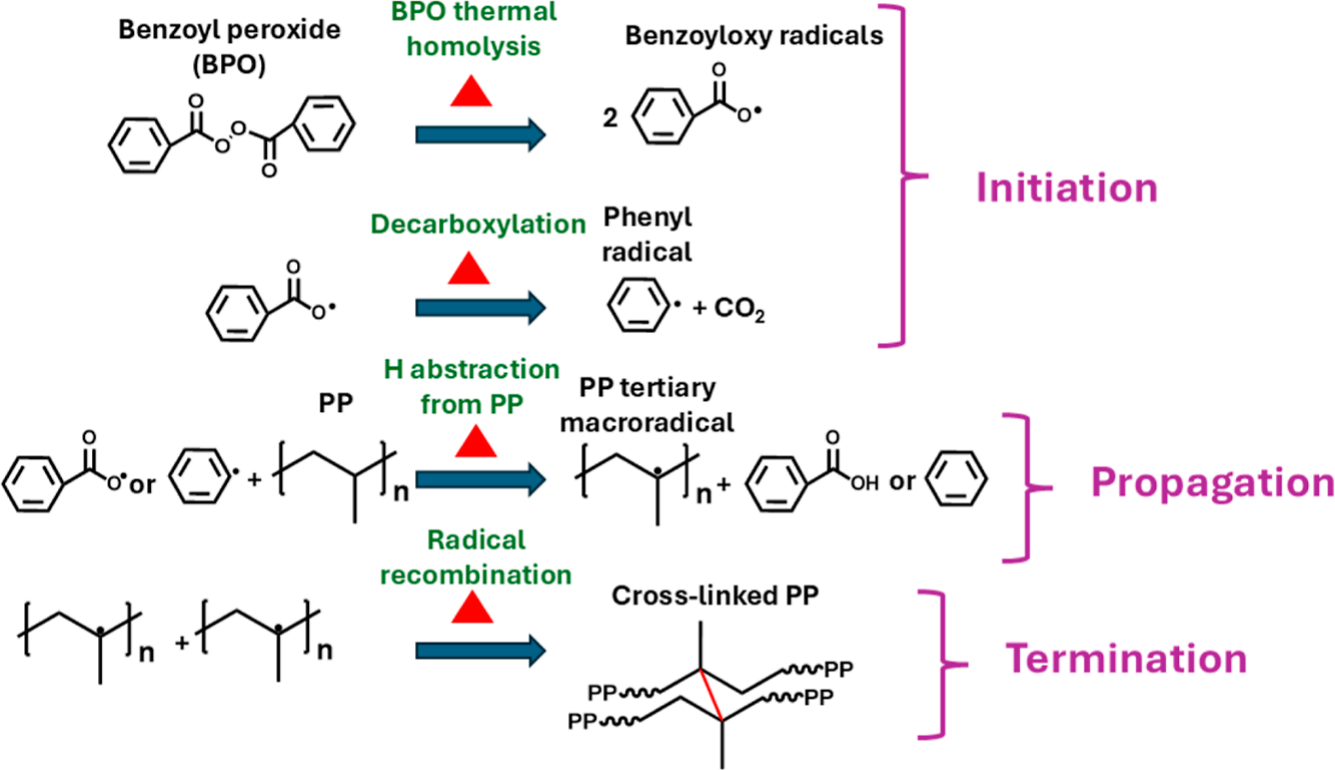
Proposed mechanism of benzoyl peroxide-induced radical formation and cross-linking in polypropylene.

This process yields PP tertiary macroradicals, which are the reactive intermediates required for the formation of chemical cross-links. Because the polymer remains below or just at the onset of melting, the material is predominantly semicrystalline, and molecular mobility is extremely restricted. Radical generation, therefore, occurs primarily in the amorphous microdomains that have sufficient local free volume to allow hydrogen abstraction.

A key feature of the solid-state environment and the central novelty of this approach is that β-scission is effectively suppressed. In molten polypropylene, PP macroradicals have sufficient conformational freedom to undergo β-scission, thereby cleaving chains and degrading the polymer. However, in the solid state, the chains are conformationally locked and cannot adopt the required geometries for C–C bond cleavage. The steric and topological constraints imposed by the crystalline lamellae prevent the backbone alignment needed for β-scission transition states, eliminating chain fragmentation as a viable pathway. As a result, all generated macroradicals are forced toward constructive radical recombination rather than destructive chain scission.

As shown in the final step of Figure 4, two immobile PP macroradicals recombine to form a new covalent C–C bond, producing either long-chain branching or true chemical cross-links between chains. Because β-scission is blocked, recombination becomes the dominant energetic pathway, significantly improving cross-linking efficiency and enabling the formation of a robust three-dimensional network without thermal degradation.

Finally, a limited amount of volatile aromatic byproducts, such as phenol, anisole, and quinone-type fragments, can be produced through secondary oxidation of the benzoyloxy and phenyl radicals. The formation of these low-molecular-weight aromatics is supported by the TGA, which shows a small mass-loss event occurring below 200 °C, consistent with the evaporation of volatile oxidation products.

## 4 Conclusion

This study demonstrates that polypropylene can be covalently cross-linked in the solid state using a simple, industrially accessible extrusion process, enabling selective chemical modification while preserving backbone integrity. The semicrystalline environment plays a decisive mechanistic role: radicals generated from BPO decompose within amorphous regions, but restricted chain mobility in the solid state suppresses β-scission and directs macroradicals toward constructive recombination. As a result, cross-linking proceeds efficiently without chain degradation, oxidative discoloration, or molecular weight loss, as is typically associated with melt-state functionalization.

Across all analytical techniques, the PP backbone remains chemically intact. FTIR and ^13^C CP-MAS NMR reveal only initiator-derived oxidation products, not PP oxidation. TGA confirms that the main degradation step and *T*_max_ remain essentially unchanged, whereas early mass loss arises from volatile BPO fragments. The modest increase in char residue with increasing initiator content is consistent with the formation of a lightly cross-linked, more thermally persistent network. DSC results show elevated crystallization temperatures and slightly reduced crystallinity, reflecting network-induced nucleation and constrained recrystallization.

Particularly, rheological characterization provides direct evidence of significant changes in the viscoelastic behavior of the modified polypropylene. The progressive increase in G′ with increasing peroxide concentration, together with the transition from crossover behavior in neat polypropylene to the dominance of G′ over G″ across the entire frequency range for PP-4 and PP-6, demonstrates the development of a network-like structure and increasing molecular connectivity. These rheological observations are consistent with the formation of intermolecular junctions via peroxide-induced radical recombination and provide independent evidence of network formation during solid-state processing.

Compared with conventional peroxide-assisted melt reactive extrusion, the present solid-state methodology achieves significant increases in melt elasticity and network formation while preserving the thermal stability and chemical structure of the polypropylene backbone. These differences highlight the unique role of the semicrystalline solid-state environment in directing radical reactions toward constructive recombination rather than degradation pathways.

Our findings establish solid-state reactive extrusion as a chemically selective, energy-efficient, and highly scalable route to modify polypropylene. This strategy opens new possibilities for high-performance PP-based materials, compatibilization and repair of recycled PP, and the development of advanced polyolefin networks while remaining fully compatible with existing industrial infrastructure.
